# Quantitative MRI Susceptibility: Mapping of Transient Ischemic Attack—A Preliminary Study

**DOI:** 10.3390/jimaging12080387

**Published:** 2026-08-17

**Authors:** Philipp Gruber, Michael Diepers, Markus Gschwind, Paul G. Unschuld, Luca Remonda, Franca Wagner, Pasquale Mordasini, Jatta Berberat

**Affiliations:** 1 Department of Diagnostic and Interventional Neuroradiology, Kantonsspital Aarau, 5001 Aarau, Switzerland; 2 Department of Neurology, Kantonsspital Aarau, 5001 Aarau, Switzerland; 3 Department of Clinical Neurosciences, University Hospitals of Geneva (HUG), 1211 Geneva, Switzerland; 4 Division of Geriatric Psychiatry, University Hospitals of Geneva (HUG), 1211 Geneva, Switzerland; 5 Department of Psychiatry, University of Geneva (UniGE), 1211 Geneva, Switzerland; 6 Department of Otorhinolaryngology, Head and Neck Surgery, Inselspital, Bern University Hospital, University of Bern, 3010 Bern, Switzerland

**Keywords:** transient ischemic attack, quantitative susceptibility mapping, MRI

## Abstract

A transient ischemic attack (TIA) is a transient episode of neurological dysfunction without evidence of acute infarct demarcation on neuroimaging. The radiographic features of TIAs are uncertain, as since there is currently no perfect imaging method with which to differentiate a stroke from a TIA. We aimed to evaluate whether regional abnormalities are associated with TIA by identifying tissue with increased susceptibility (χ) values using quantitative susceptibility mapping (QSM). A total of 39 adults with clinical TIA symptoms (66 ± 12 years) and 39 age- and sex matched controls (65 ± 12 years) underwent MRI. Based on the QSM-maps, local susceptibility changes were tested for statistically significant differences between patients with TIA and healthy controls. The susceptibility maps of TIA patients showed that magnetic susceptibility differed from that of healthy volunteers, with the strongest effects observable in the right lingual gyrus (*p* < 0.001) and bilaterally in the caudal anterior cingulate (cACC, *p* < 0.01). TIA is often difficult to diagnose at the time of presentation in the emergency department, and QSM could show an association between regional QSM abnormalities and TIA.

## 1. Introduction

A transient ischemic attack (TIA) is a transient episode of neurological dysfunction, without evidence of acute infarction on neuroimaging. In the past, TIA was arbitrarily distinguished from stroke by a duration of neurological symptoms of less than 24 h [1]. However, this definition allowed for patients at risk of permanent infarction to be misclassified as having a TIA, and thus the aforementioned tissue-based definition is favored [1]. It is well established that symptoms of TIAs usually resolve within 24 h, often within hours, but prolonged episodes occasionally occur [1,2].

There is uncertainty regarding the radiographic features of TIAs since there is no perfect imaging method with which to differentiate a stroke from a TIA [1]. Whereas CT scans of the brain are often unremarkable, there is controversy regarding the significance of regions demonstrating high signal intensity on DWI, a finding present in approximately half of all TIAs [3]. Therefore, additional imaging markers are desirable for the diagnosis of TIA.

A TIA is characterized by a temporary disturbance of brain function in a specific region, caused by a transient reduction in cerebral blood flow. Stroke symptoms are often identical to those of TIA initially, but they do not resolve on their own and can result in lasting neurological deficits depending on their severity and the area of the brain affected.

Magnetic susceptibility differences in the brain can be measured by MRI using quantitative susceptibility mapping (QSM). QSM uses the magnetization induced in tissue when placed in a magnetic field to provide a quantitative measure of the tissue susceptibility (χ) distribution [4,5]. QSM improves the detection and identification of iron and calcium deposition and allows better characterization of pathological iron and myelin variations, as well as unique details of brain morphology within deep brain nuclei [6,7]. Several QSM-MRI studies have shown an association between altered tissue magnetic susceptibility and various conditions such as attention deficit/hyperactivity disorder [8], multiple sclerosis [9,10], Huntington’s disease [11,12], Parkinson’s disease [13,14], and Alzheimer’s disease [11,15,16,17]. QSM has also been extensively evaluated for application to ischemic stroke [18,19].

Higher magnetic susceptibility in cortical veins has been observed on susceptibility maps in the ischemic hemisphere of stroke patients, indicating an increased oxygen extraction fraction [19,20,21,22]. As susceptibility reflects the magnetizability of a tissue, it is sensitive to local and global oxygenation. Thus, magnetic susceptibility-based contrast has been used to determine oxygenation status in the brain [22,23,24,25]. QSM has been applied in the assessment of vessel function and oxygen metabolism in patients and animal models with acute ischemic stroke [19,26,27,28,29]. In patients with acute stroke, prominent vessels with high magnetic susceptibility were observed with SWI and QSM [28,30,31,32,33]. To the best of our knowledge, no studies on the application of QSM to TIA have so far been published.

We aimed to evaluate whether regional QSM abnormalities are associated with TIA, potentially providing an imaging marker with which to identify transiently hypoperfused tissue by detecting microstructural alterations in tissue susceptibility. For this, we compared the χ values of TIA patients with those of a group of age- and sex-matched healthy volunteers to identify possible underlying alterations in regional susceptibility values.

## 2. Materials and Methods

### 2.1. Subjects

We recruited 39 patients (age > 18 years) who presented with stroke-like symptoms at the emergency department of our hospital (sudden weakness or numbness on one side of the body, difficulty speaking or understanding speech, vision problems, dizziness and severe headache, as well as vision loss, blurred vision or double vision, vertigo, confusion, and problems with balance and coordination). All these patients had unremarkable results of examination of the brain with CT angiography and CT perfusion. All patients underwent an additional MRI scan for further clarification of the pathology underlying the clinical symptoms within 24 h of enrollment in the study. Each patient was informed about the study prior to the MRI, and participation was voluntary. Informed consent was given by all participants prior to enrollment in the study. The study was approved by the local ethics committee (EKNZ 2022-01947).

In addition, 39 age- and sex-matched healthy volunteers were scanned (age > 18 years). All potential participants with contraindications to MRI [34] or a known history of cerebrovascular diseases were excluded from the study.

After a board-certified neuroradiologist had screened the images and excluded other possible reasons for the stroke-like symptoms, a final diagnosis of TIA was made by the neurological senior supervisor during hospitalization of the patient. Diagnosis was based on a full neurologic examination. In addition, an electrocardiogram was performed to evaluate for atrial fibrillation and blood pressure, pulse rate, and oxygen saturation were measured, as well as routine blood work.

### 2.2. Mri

All MRIs were acquired on a 3 Tesla MRI Scanner (Skyra or Vida, Siemens Healthineers, Erlangen, Germany). For image acquisition, a 32-head channel coil was used. Our in-house stroke protocol was followed, which included the following sequences: DWI, SWI and T2-weighted FLAIR, followed by the QSM sequence (TR = 59 msec, 8 TEs = 5, 8, 11, 14, 17, 20, 23, 26 msec, flip angle = 20°, FOV = 220 × 220 mm^2^, slice thickness = 2 mm, 1 average, TA = 6:27 min) and anatomical T1 MP-RAGE sequence (TR = 2230 msec, TE = 4.78 msec, flip angle = 9°, FOV = 220 × 220 mm^2^, slice thickness = 0.9 mm, 1 average, TA = 5:05 min).

### 2.3. Image Analysis

The QSM maps were obtained using the QSMxT framework [35]. The phase unwrapping, background field removal, and dipole inversion steps were achieved using the TGV-QSM algorithm. T1-weighted images were segmented using FreeSurfer via the Aseg probabilistic atlas. Then, the T1-weighted anatomical images were registered to the magnitude images in the QSM space using the MINC toolkit. QSM statistics for 46 brain regions were extracted.

### 2.4. Statistics

Statistical analysis was performed using SPSS statistics software (version 29) (IBM Corporation, Armonk, NY, USA). An unpaired *t*-test together with Cohen’s d absolute effect size was used to test statistically significant differences in normally distributed QSM values between TIA patients and healthy volunteers. *p*-values were indicated after controlling the false discovery rate using the Benjamini–Hochberg procedure to correct for performing 46 tests [36]. Pearson’s correlation coefficient was calculated between the imaging delay and susceptibility values in different brain areas. Results are expressed as mean ± SD. Values outside the range of mean ± 4 × SD were excluded from the analysis.

## 3. Results

Demographic information and the results of clinical tests on all participants are summarized in Table 1. No statistically significant differences in demographic characteristics (age, weight, BMI and sex) were detected between the patients with TIA and healthy volunteers. All the patients were asymptomatic within 24 h (15 ± 10 h, ranging from minutes to 23 h). Symptom duration varied from a couple of seconds to several hours, up to 1 day. One patient had a focal DWI hyperintensity without any correlation on the corresponding ADC map. The FLAIR image confirmed it to be a classical “T2 shine-through” effect. The median time delay until MRI was conducted was 21 h (17 ± 7 h, range 2 to 23 h). No correlation was detected between the imaging delay and the susceptibility values of the TIA individuals.

**Figure 1:**
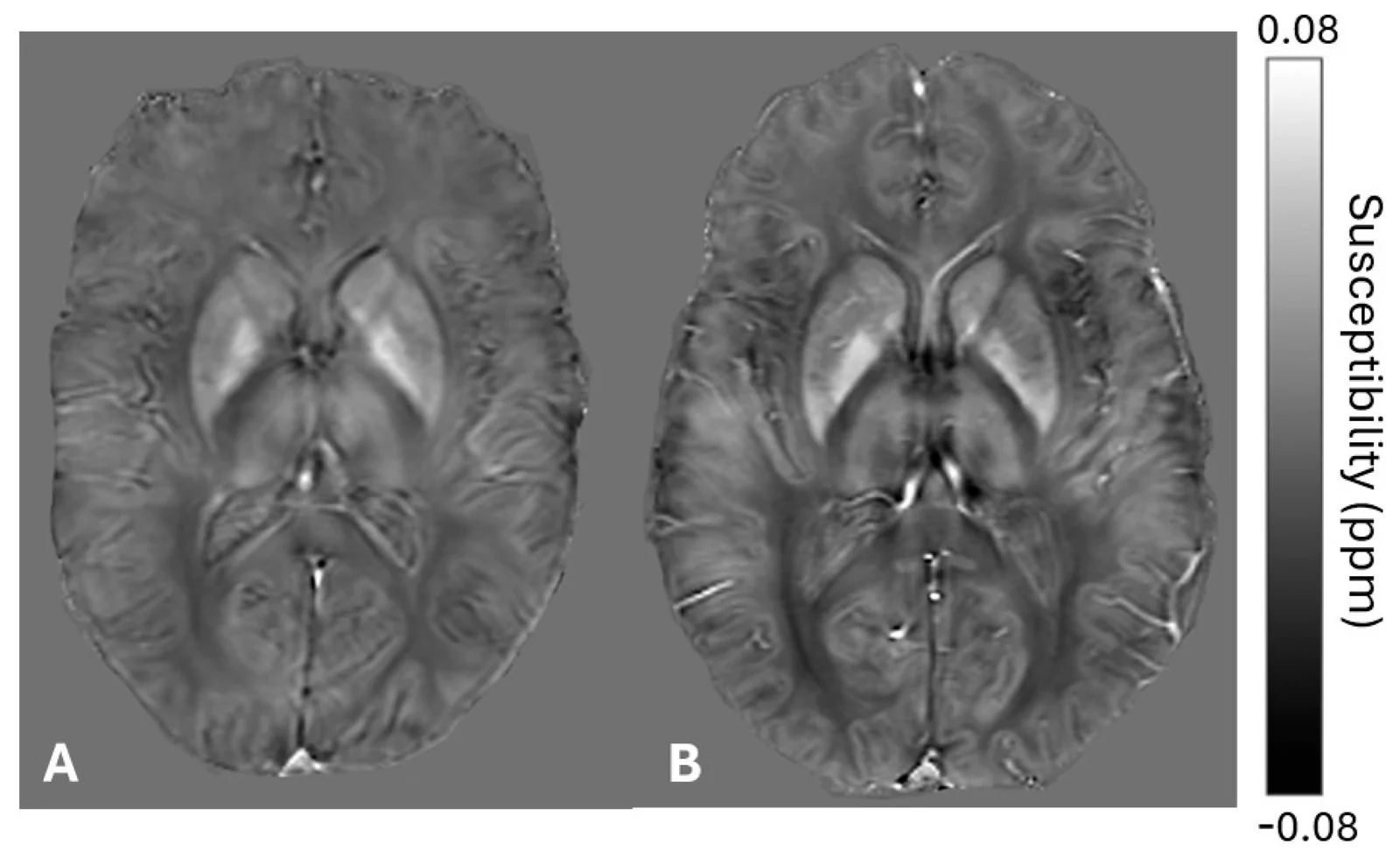
Susceptibility maps indicating the regional distribution of brain tissue susceptibility: (**A**) healthy control and (**B**) individual with TIA.

**Figure 2:**
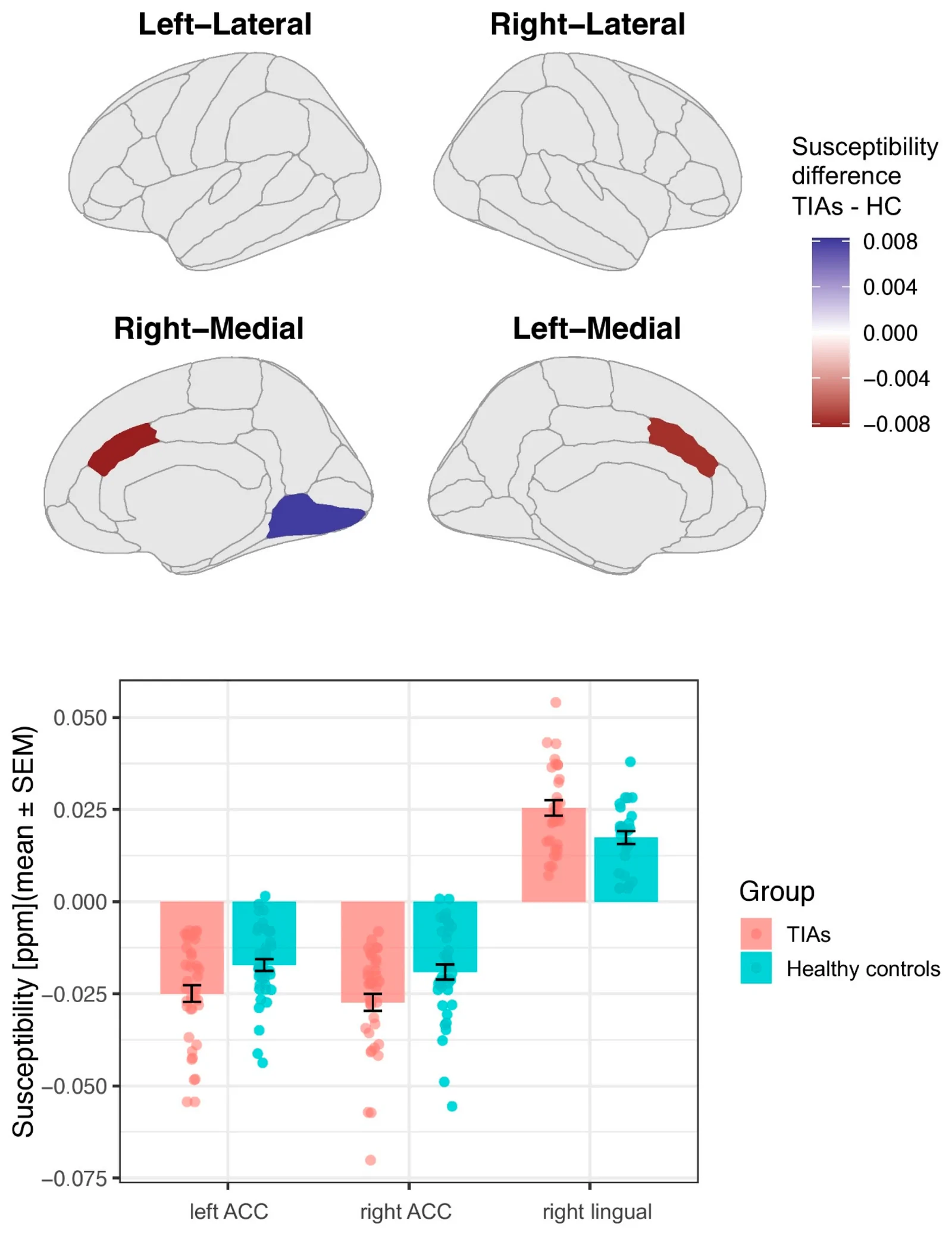
Areas where susceptibility values showed statistically significant differences in persons with TIA compared to healthy volunteers: the right lingual gyrus (*p* < 0.001) and bilaterally in the caudal anterior cingulate cortex (cACC, *p* < 0.01).

Figure 1 shows examples of susceptibility maps, indicating the regional distribution of brain tissue susceptibility, for a healthy control and an individual with TIA. To detect differences in mean ROI susceptibility between controls and patients with TIA, 46 *t*-tests were performed (see Supplementary Materials). The resulting *p*-values were adjusted for multiple testing by using the Benjamini–Hochberg false discovery rate control [36], resulting differences in susceptibility levels between controls and TIA patients in the right lingual gyrus (*p* < 0.001, d = −0.766) and bilateral caudal anterior cingulate (cACC, *p* < 0.01, d(R) = 0.614 and d(L) = 0.635, respectively) (Table 1, Figure 2).

## 4. Discussion

In this study, we used QSM to assess magnetic abnormalities indicating local alterations of an increased oxygen extraction fraction inducing an increase in paramagnetic deoxyhemoglobin due to hypoperfusion. Compared to healthy volunteers, susceptibility values in persons with TIA showed statistically significant differences in the right lingual gyrus and bilateral cACC.

TIA is associated with time-limited brain dysfunction in a circumscribed area. This is caused by a regional reduction in cerebral blood flow, resulting in either transient or minor observable clinical symptoms.

Areas exhibiting statistically significant differences in tissue susceptibility demonstrate a noteworthy correlation with brain regions known to manifest symptoms following a TIA, suggesting a potential link between regional neurological dysfunction and an increased oxygen extraction fraction [22,37].

It is known that brain iron concentrations increase with normal aging [38]. However, elevated iron levels may also result from a variety of conditions, including neurodegenerative diseases [38]. Recent studies suggest that serum ferritin levels could be a novel predictor of hemorrhagic transformation following ischemic stroke. Experimental investigations have confirmed the detrimental impact of iron overload on the ischemic brain and the endothelial lining of blood vessels [39].

During healthy aging, iron deposits accumulate heterogeneously in specific regions of the brain and are largely located in the deep gray matter nuclei [6,40]. There is a rapid increase in iron accumulation from birth up to the age of 20 years; it slows down in middle age and increases again after 60 years [6,41]. Our cohorts were therefore strictly age-matched to avoid any bias due to normal aging.

We acknowledge several limitations of our study. The comparison between TIA patients and healthy controls did not include minor strokes. However, affected patients can normally be accurately diagnosed based on routine MRI and/or CT images. Furthermore, acquisition time increased due to the QSM sequence, which could be challenging in an emergency clinical setting. The number of subjects recruited in our study was relatively small, and several patients suffered from overlapping symptoms; it was not possible to divide patients into subgroups based on their symptoms. The findings in the lingual gyrus and cACC may reflect broader microvascular/perfusion vulnerability rather than focal symptom-causing lesions. Therefore, these results should be considered preliminary, and further studies are needed to confirm our findings—these findings cannot yet support diagnostic use. Only healthy controls were included in the study, since clinical MRI sequences do not offer any possibility to differentiate TIAs from controls. There is a possibility that QSM abnormalities are not only unique to TIA but reflect underlying cerebrovascular disease burden; however, this is already enough to differentiate them from other possible reasons for stroke-like symptoms. Our healthy controls were recruited without a known history of cerebrovascular diseases. However, additional factors possibly influencing susceptibility values, such as hypertension, diabetes, smoking, renal dysfunction, medications, and anemia, were not examined in the healthy control group. Detailed matching for systemic metabolic risk factors was outside our study scope.

Early assessment of patients arriving at the emergency department makes the diagnosis of TIA enormously difficult. The diagnosis of TIA depends on the quality and quantity of information available and the time of assessment. Imaging-sensitive markers would be invaluable for making the correct diagnosis of TIA.

As many as 60% of patients referred to a TIA clinic will not have a final diagnosis of TIA [42,43]. Identification of possible TIA mimics is an important stage in the assessment of patients with transient neurologic symptoms. An accurate diagnosis of a stroke mimic impacts treatment decisions and provides reassurance when the diagnosis turns out to be less serious (for example, TIA).

## 5. Conclusions

Early clinical assessment in the emergency setting often presents challenges in diagnosing TIA. QSM suggests an association between regional QSM abnormalities and TIA and possibly has promise in the future as an imaging biomarker, thereby enhancing diagnostic precision, informing timely treatment decisions, and improving patient reassurance.

## Figures and Tables

**Table 1:** Pooled data of all the study subjects (mean ± SD).

	Healthy Controls (*n* = 39)	TIA (*n* = 39)
Sex (f/m) *	22/17	19/20
Age (years)	65 ± 12	66 ± 12
Weight (kg)	74 ± 13	76 ± 13
Height (cm)	170 ± 9	168 ± 7
BMI (kg/m^2^) **	26 ± 4	27 ± 4
Clinical symptoms (one patient can have several symptoms)	*n.a.*	
Sudden weakness or numbness on one side of the body, problems to move *n* (%)		(17) 44%
Difficulty in speaking *n* (%)		(10) 23%
Difficulty of understanding speech *n* (%)		(1) 3%
Visual disturbances *n* (%)		(10) 26%
Vertigo *n* (%)		(15) 38%
Disorientation *n* (%)		(5) 13%
Problems with balance and coordination *n* (%)		(5) 13%
Headache *n* (%)		(2) 5%
Symptom duration		
<24 h *n* (%)	*n.a.*	39 (100%)
Diffusion restriction *n* (%)	*n.a.*	(1) 3%
χ (ppm)	HC	TIA
R lingual gyrus (*p* < 0.001, d = −0.766)	0.0174 ± 0.0091	0.0254 ± 0.0116
R ACC (*p* < 0.01, d = 0.614)	−0.0191 ± 0.0128	−0.0273 ± 0.0139
L ACC (*p* < 0.01, d = 0.635)	−0.0172 ± 0.0102	−0.0249 ± 0.0138

* f = female; m = male, ** BMI = Body Mass Index; R = right; L = left; ACC = caudal anterior cingulate; χ = susceptibility, ppm = parts per million; d = Cohen’s d absolute effect size; *n.a. = not applicable*.

## Data Availability

The data presented in this study are available on request from the corresponding author due to privacy or ethical restrictions.

## Supplementary Materials

The following supporting information can be downloaded at https://www.mdpi.com/article/10.3390/jimaging12080387/s1, Table S1: Additional brain areas tested for a statistically significant difference between patients with TIAs and healthy controls.

## Abbreviations

cACC	Caudal anterior cingulate
QSM	Quantitative susceptibility mapping
TIA	Transient ischemic attack
