# Supplementary material for: Quantitative MRI Susceptibility: Mapping of Transient Ischemic Attack—A Preliminary Study
Source: J Imaging. 2026 Aug 17;12(8):387. doi: 10.3390/jimaging12080387 (PMC13514822; doi:10.3390/jimaging12080387)
Supplement: Supplementary file 1 [file jimaging-12-00387-s001.zip › jimaging-4489311-supplementary.pdf]

Journal of Imaging

MDPI

Supplemental materials

Quantitative MRI Susceptibility: Mapping of Transient Ischemic Attack—A Preliminary Study

Philipp Gruber [1], Michael Diepers [1], Markus Gschwind [2,3], Paul G. Unschuld [4,5], Luca Remonda [1], Franca Wagner [1,6], Pasquale Mordasini [1] and Jatta Berberat [1,*]

1 Department of Diagnostic and Interventional Neuroradiology, Kantonsspital Aarau, 5001 Aarau, Switzerland
2 Department of Neurology, Kantonsspital Aarau, 5001 Aarau, Switzerland
3 Department of Clinical Neurosciences, University Hospitals of Geneva (HUG), 1211 Geneva, Switzerland
4 Division of Geriatric Psychiatry, University Hospitals of Geneva (HUG), 1211 Geneva, Switzerland
5 Department of Psychiatry, University of Geneva (UniGE), 1211 Geneva, Switzerland
6 Department of Otorhinolaryngology, Head and Neck Surgery, Inselspital, Bern University Hospital, University of Bern, 3010 Bern, Switzerland
* Correspondence: jatta.berberat@ksa.ch; Tel.: +41-(0)-62-838-43-21; Fax: +41-(0)-62-838-52-23

Table S1. Additional brain areas tested for a statistically significant difference between patients with TIAs and healthy controls.

χ (ppm)	Healthy controls (n = 39)	TIA (n = 39)	*P, d*
R Hippocampus	0.0226 ± 0.010	0.0267 ± 0.0135	0.1316, 0.0345
L Hippocampus	0.0251 ± 0.0104	0.0272 ± 0.0145	0.4646, −0.166
R Pallidum	0.0975 ± 0.0183	0.1023 ± 0.0209	0.284, −0.244
L Pallidum	0.0966 ± 0.0224	0.0975 ± 0.0224	0.860, −0.040
R Putamen	0.0396 ± 0.0177	0.0460 ± 0.0158	0.096, −0.381
L Putamen	0.0519 ± 0.0226	0.0560 ± 0.0233	0.433, −0.179
R Thalamus-Proper	0.0106 ± 0.0110	0.0082 ± 0.0153	0.429, 0.180
L Thalamus-Proper	0.0104 ± 0.0120	0.0090 ± 0.0170	0.677, 0.095
R Ventral DC	0.0076 ± 0.0160	0.0092 ± 0.0194	0.693, −0.090
L Ventral DC	0.0105 ± 0.0116	0.0102 ± 0.0189	0.934, 0.019
R Choroid Plexus	0.0113 ± 0.0109	0.0101 ± 0.0160	0.700, 0.088
L Choroid Plexus	0.0033 ± 0.0144	0.0014 ± 0.0173	0.597, 0.120
R Nucleus Accumbens	−0.0034 ± 0.0208	−0.0095 ± 0.0175	0.170, 0.317
L Nucleus Accumbens	−0.0086 ± 0.0191	−0.0102 ± 0.0158	0.688, 0.091
R Amygdala	0.0201 ± 0.0119	0.0221 ± 0.0149	0.514, −0.148
L Amygdala	0.0208 ± 0.0142	0.0279 ± 0.0212	0.088, −0.394
R Caudate	0.0387 ± 0.0099	0.0413 ± 0.0129	0.321, −0.226
L Caudate	0.0430 ± 0.0119	0.0429 ± 0.0112	0.970, 0.009
R Caudal Middle Frontal Gyrus	0.0014 ± 0.0069	0.0024 ± 0.0086	0.573, −0.128
L Caudal Middle Frontal Gyrus	0.0031 ± 0.0062	0.0035 ± 0.0057	0.769, −0.067
R Cuneus	−0.0044 ± 0.0088	−0.0029 ± 0.0074	0.418, −0.184
L Cuneus	−0.00008 ± 0.0060	−0.00244 ± 0.0087	0.171, 0.315
R Insula	−0.0174 ± 0.0086	−0.0146 ± 0.0093	0.173, −0.313
L Insula	−0.0130 ± 0.0089	−0.0153 ± 0.0096	0.276, 0.248
R Isthmus Cingulate Gyrus	−0.0126 ± 0.0118	−0.0166 ± 0.0300	0.441, 0.175
L Isthmus Cingulate Gyrus	−0.0103 ± 0.0136	−0.0157 ± 0.0280	0.281, 0.246
R Paracentral Lobule	0.0236 ± 0.0228	0.0166 ± 0.0243	0.198, 0.297
L Paracentral Lobule	0.0255 ± 0.0272	0.0133 ± 0.0259	0.051, 0.459

R Pars Opercularis	−0.0083 ± 0.0077	−0.0088 ± 0.0073	0.769, 0.067
L Pars Opercularis	−0.0134 ± 0.0074	−0.0150 ± 0.0084	0.375, 0.202
R Pars Triangularis	0.0057 ± 0.0091	0.0048 ± 0.0072	0.627, 0.110
L Pars Triangularis	0.0046 ± 0.0084	0.0011 ± 0.0079	0.060, 0.430
R Peri Calcarine Cortex	0.0175 ± 0.0109	0.0243 ± 0.0225	0.088, −0.385
L Peri Calcarine Cortex	0.0181 ± 0.0099	0.0245 ± 0.042	0.354, −0.211
R Posterior Cingulate Cortex	−0.0018 ± 0.0115	−0.0004 ± 0.0172	0.678, −0.096
L Posterior Cingulate Cortex	−0.0034 ± 0.0117	−0.0016 ± 0.0157	0.565, −0.130
R Precuneus	0.0011 ± 0.0092	0.0016 ± 0.0145	0.854, −0.041
L Precuneus	−0.00009 ± 0.01697	0.00002 ± 0.01012	0.972, −0.008
R Rostral Anterior Cingulate	−0.0014 ± 0.0072	−0.0033 ± 0.0055	0.194, 0.297
L Rostral Anterior Cingulate	0.0010 ± 0.0068	−0.0010 ± 0.0064	0.194, 0.303
R Transverse Temporal Gyrus	0.0202 ± 0.0122	0.0263 ± 0.0153	0.057, −0.441
L Transverse Temporal Gyrus	0.0266 ± 0.0121	0.0298 ± 0.0176	0.359, −0.212
L Lingual Gyrus	0.0164 ± 0.0108	0.0203 ± 0.0115	0.131, −0.350

R = right; L = left; χ = susceptibility; ppm = parts per million; DC = ventral part of the diencephalon, including the thalamus and hypothalamus, excluding the anterior commissure and optic chiasm, extending laterally to the internal capsule; *P* = corrected *p*-value after the multiple test correction; *d* = Cohen's d absolute effect size.

https://doi.org/10.3390/jimaging12080387
